# Multicenter Study of Trimethoprim/Sulfamethoxazole-Related Hepatotoxicity: Incidence and Associated Factors among HIV-Infected Patients Treated for *Pneumocystis jirovecii* Pneumonia

**DOI:** 10.1371/journal.pone.0106141

**Published:** 2014-09-03

**Authors:** Jen-Jia Yang, Chung-Hao Huang, Chun-Eng Liu, Hung-Jen Tang, Chia-Jui Yang, Yi-Chien Lee, Kuan-Yeh Lee, Mao-Song Tsai, Shu-Wen Lin, Yen-Hsu Chen, Po-Liang Lu, Chien-Ching Hung

**Affiliations:** 1 Department of Internal Medicine, National Taiwan University Hospital and National Taiwan University College of Medicine, Taipei, Taiwan; 2 Department of Internal Medicine, Po Jen General Hospital, Taipei, Taiwan; 3 Department of Internal Medicine, Kaohsiung Medical University Hospital and Kaohsiung Medical University, Kaohsiung, Taiwan; 4 Department of Internal Medicine, Changhua Christian Hospital, Changhua, Taiwan; 5 Department of Internal Medicine, Chi Mei Medical Center, Tainan, Taiwan; 6 Department of Internal Medicine, Far Eastern Memorial Hospital, New Taipei City, Taiwan; 7 Department of Internal Medicine, Ditmanson Medical Foundation Chia-Yi Christian Hospital, Chia-Yi, Taiwan; 8 Department of Internal Medicine, National Taiwan University Hospital Hsin-Chu Branch, Hsin-Chu, Taiwan; 9 Graduate Institute of Clinical Pharmacy, National Taiwan University, Taipei, Taiwan; 10 Department of Pharmacy, National Taiwan University Hospital and National Taiwan University College of Medicine, Taipei, Taiwan; 11 Department of Medical Research, China Medical University Hospital, Taichung, Taiwan; 12 China Medical University, Taichung, Taiwan; University of British Columbia, Canada

## Abstract

The incidence of hepatotoxicity related to trimethoprim/sulfamethoxazole (TMP/SMX) administered at a therapeutic dose may vary among study populations of different ethnicities and hepatotoxic metabolites of TMP/SMX may be decreased by drug-drug interaction with fluconazole. We aimed to investigate the incidence of hepatotoxicity and the role of concomitant use of fluconazole in HIV-infected patients receiving TMP/SMX for *Pneumocystis jirovecii* pneumonia. We reviewed medical records to collect clinical characteristics and laboratory data of HIV-infected patients who received TMP/SMX for treatment of *P. jirovecii* pneumonia at 6 hospitals around Taiwan between September 2009 and February 2013. Hepatotoxicity was defined as 2-fold or greater increase of aminotransferase or total bilirubin level from baselines. Roussel UCLAF Causality Assessment Method (RUCAM) was used to analyze the causality of drug-induced liver injuries. *NAT1* and *NAT2* acetylator types were determined with the use of polymerase-chain-reaction (PCR) restriction fragment length polymorphism to differentiate common single-nucleotide polymorphisms (SNPs) predictive of the acetylator phenotypes in a subgroup of patients. During the study period, 286 courses of TMP/SMX treatment administered to 284 patients were analyzed. One hundred and fifty-two patients (53.1%) developed hepatotoxicity, and TMP/SMX was considered causative in 47 (16.4%) who had a RUCAM score of 6 or greater. In multivariate analysis, concomitant use of fluconazole for candidiasis was the only factor associated with reduced risk for hepatotoxicity (adjusted odds ratio, 0.372; 95% confidence interval, 0.145–0.957), while serostatus of hepatitis B or C virus, *NAT1* and *NAT2* acetylator types, or receipt of combination antiretroviral therapy was not. The incidence of hepatotoxicity decreased with an increasing daily dose of fluconazole up to 4.0 mg/kg. We conclude that the incidence of TMP/SMX-related hepatotoxicity was 16.4% in HIV-infected Taiwanese patients who received TMP/SMX for pneumocystosis. Concomitant use of fluconazole was associated with decreased risk for TMP/SMX-related hepatotoxicity.

## Introduction

*Pneumocystis jirovecii* pneumonia remains one of the most important causes of pulmonary opportunistic infections in HIV-infected patients in the era of combination antiretroviral therapy (cART), accounting for more than half of the pulmonary complications in patients whose CD4 lymphocyte count is less than 200 cells/µL [1], [2]. The mortality rate of *P. jirovecii* pneumonia is approximately 10% to 12% in the HIV-infected patients [3]. While trimethoprim/sulfamethoxazole (TMP/SMX) has been the recommended treatment of choice for *P. jirovecii* pneumonia in HIV-infected patients, TMP/SMX administered at therapeutic doses may cause several adverse effects such as allergy, hepatotoxicity, bone marrow suppression, hyperkalemia, and nephrotoxicity [4], [5]. Use of TMP/SMX in treatment of *P. jirovecii* pneumonia has been recently shown to cause acute psychosis in HIV-infected patients and transplant recipients and the incidence increases with the dose administered [4]–[7].

Hepatotoxicity has been reported to occur in less than 10% of the patients who received TMP/SMX in treatment of pneumocystosis in clinical trials, and most cases of hepatotoxicity occurred on the 2^nd^ to 12^th^ day after initiation of TMP/SMX [8]. Two mechanisms have been proposed for TMP/SMX-related hepatotoxicity: allergic response and metabolite-related toxicity. For the latter mechanism, the hepatotoxic metabolite of TMP/SMX, hydroxylamine, is produced after TMP/SMX enters the metabolic pathway through cytochrome protein 450 (CYP450) subtype 2C9 in the liver [9], [10]. There are two major patterns of hepatotoxicity: hepatocellular and cholestatic, with the latter being more commonly reported in previous studies [9], [11]–[14].

The occurrence of hepatotoxicity may vary with the pharmacogenetics of different ethnicities enrolled in the studies, and concurrent use of multiple medications with drug-drug interactions may pose challenges in determining the culprit of hepatotoxicity. For example, concomitant use of fluconazole for oro-esophageal candidiasis is common in HIV-infected patients who present with *P. jirovecii* pneumonia, which may raise concerns because fluconazole may potentially increase the risk of hepatotoxicity when combined with TMP/SMX. In this multicenter study, we aimed to investigate the incidence of hepatotoxicity with the use of Roussel UCLAF Causality Assessment Method (RUCAM) scale [15]; to identify its associated factors; and to investigate the role of concomitant use of fluconazole in hepatotoxicity in HIV-infected adult patients who received TMP/SMX in the treatment of *P. jirovecii* pneumonia at referral hospitals for HIV care around Taiwan.

## Materials and Methods

### Study setting and population

This multicenter, retrospective cohort study was conducted at 6 hospitals designated for HIV care around Taiwan, where inpatient or outpatient HIV care, including cART and monitoring of plasma HIV RNA load and CD4 count, are provided free-of-charge. We reviewed the medical records of the HIV-infected patients, aged 20 years or greater, who presented with *P. jirovecii* pneumonia and received TMP/SMX from July 2009 to February 2013. The cases of *P. jirovecii* pneumonia were identified from the computerized databases of the participating hospitals. The diagnosis of *P. jirovecii* pneumonia was made based on identification of *P. jirovecii* by Giemsa stain of the sputum or bronchoalveolar lavage specimens, or histopathology of transbronchial or surgical lung biopsy specimens; detection of *Pneumocystis* 16S rRNA in the sputum or bronchoalveolar lavage specimens plus a typical clinical history and chest radiography that was consistent with interstitial pneumonitis; or a typical clinical history and computed tomography of the chest consistent with interstitial pneumonitis plus clinical response to TMP/SMX or clindamycin plus primaquine. The patients who did not have at least 1 follow-up liver function value within 1 month after initiation of TMP/SMX or had no baseline liver function value were excluded. The study was approved by the Research Ethics Committees of the participating hospitals (registration no. 201205032RIB) and written or oral informed consent was waived. The health records of the patients were anonymized prior to use.

### Data collection

A standardized case record form was used to collect information on demographic and clinical characteristics such as age, sex, risk factors for HIV infection, smoking status, alcohol use, comorbidity (diabetes mellitus, hypertension, chronic lung disease, chronic kidney disease, malignancy, or tuberculosis), serostatus of hepatitis B and C viruses, concurrent medications including steroids, antimicrobials, and cART before and during the treatment course of TMP/SMX, weight and body-mass index (BMI) (expressed in kilogram divided by height in square meters), dose and treatment duration of TMP/SMX, complications of *P. jirovecii* pneumonia (respiratory failure or admission to the intensive care unit), and outcome.

In this retrospective cohort study, we collected all the laboratory test results that were available within 1 month before or nearest to the start of TMP/SMX (baseline) and during the treatment course from the computerized databases of the participating hospitals. The data collected included total and direct bilirubin, aspartate aminotransferase (AST), alanine aminotransferase (ALT), and alkaline phosphatase (ALP), CD4 count and plasma HIV RNA load, serum creatinine and electrolyte levels, and lactate dehydrogenase (LDH). The highest values of serial data of aminotransferases and bilirubin following the initiation of TMP/SMX were used to categorize the severity (grading) of hepatotoxicity.

To analyze the relationship between the acetylator genotypes and TMP/SMX-related hepatotoxicity, the available data of *NAT1* (*N*-acetyltransferase-1) and *NAT2* (*N*-acetyltransferase-2) genes were collected from 141 patients who were subsequently enrolled in a pharmacogenetic study for antiretroviral therapy at the National Taiwan University Hospital (Hung CC, unpublished data).

The data were collected by the HIV-treating physicians at the 6 participating hospitals, which were rechecked by the principal investigators (Yang JJ and Hung CC) to resolve and correct the inconsistencies and errors identified.

#### Laboratory investigations

Plasma HIV RNA load was quantified using the Cobas Amplicor HIV-1 Monitor test (Cobas Amplicor version 1.5, Roche Diagnostics Corporation, IN) with a lower detection limit of 40 copies/mL, and CD4 count was determined using FACFlow (BD FACS Calibur, Becton Dickinson, CA). Anti-hepatitis A virus antibodies were determined using chemiluminescence immunoassay (CIA) (Abbott Diagnostics: CMIA-ARCHITECT i-2000); hepatitis B surface antigen (HBsAg), anti-HBs antibody, and hepatitis B core antibody (anti-HBc antibody) using enzyme immunoassay (Abbott Laboratories, Abbott Park, IL); and antibodies to hepatitis C virus using a third-generation enzyme immunoassay (Ax SYM HCV III, Abbott Laboratories, North Chicago, IL). *NAT1* and *NAT2* acetylator types were determined with the use of polymerase-chain-reaction (PCR) restriction fragment length polymorphism to differentiate common single-nucleotide polymorphisms (SNPs) predictive of the acetylator phenotypes [16], [17].

#### Definitions of hepatotoxicity

Hepatotoxicity was defined as 2-fold or greater increase of aminotransferase or total bilirubin level from baseline based on the reports of international consensus meetings [15],[18]. RUCAM scale was used to evaluate whether the hepatotoxicity was related to the use of TMP/SMX. The RUCAM scale includes the evaluation parameters of time course, other risk factors for hepatotoxicity, concomitant medications, survey for non-drug causes, previous recorded hepatotoxicity for the drug, and response to re-administration. A score of RUCAM scale of 6 to 8 is considered as probably related, and a score of greater than 8 as highly probably related [19], [20]. Grading of hepatotoxicity was determined based on the Division of AIDS table for grading the severity of adult and pediatric adverse events (2004 version 1, 2009 clarification) [21] and the grading was determined using the ratio of the follow-up aminotransferase or total bilirubin data divided by the baseline values if the baseline liver function profiles were abnormal. The table defines grade 1 through 4 hepatotoxicity as follows: grade 1, elevation of aminotransferases by 1.25–2.5× upper limit of normal (ULN); grade 2, 2.6–5.0× ULN, grade 3, 5.1–10.0× ULN and grade 4, >10.0× ULN]; or grade 1, elevation of total bilirubin by 1.1–1.5× ULN; grade 2, 1.6–2.5× ULN; grade 3, 2.6–5.0× ULN; and grade 4, >5.0× ULN. The type of hepatotoxicity was categorized by the equation (R = [ALT/ULN (upper limit of normal)]/[ALP/ULN]) as hepatocellular (ALT≧3× ULN and R≧5), cholestatic (ALP≧2× ULN and R≦2), or mixed types (ALT≧3× ULN, ALP≧2× ULN and 2<R<5).

### Statistical analysis

All statistical analyses were performed using the statistical package SAS 9.3 (SAS Institute Inc., Cary, North Carolina, USA). The Fisher exact or the Chi square tests were used to compare categorical variables and the Wilcoxon test to compare continuous variables between the patients with TMP/SMX-related hepatotoxicity and those without hepatotoxicity. All comparisons were two-tailed and a *P* value <0.05 was considered significant. We included the variables with a *P*-value <0.1 in the univariate analysis such as BMI, diabetes mellitus, CD4 count at the start of TMP/SMX, and presence of hyperkalemia or psychosis, and variables that were of biological significance such as possible hepatotoxic agents (fluconazole or antiretroviral agents) in the multivariate logistic regression models.

## Results

**Figure 1:**
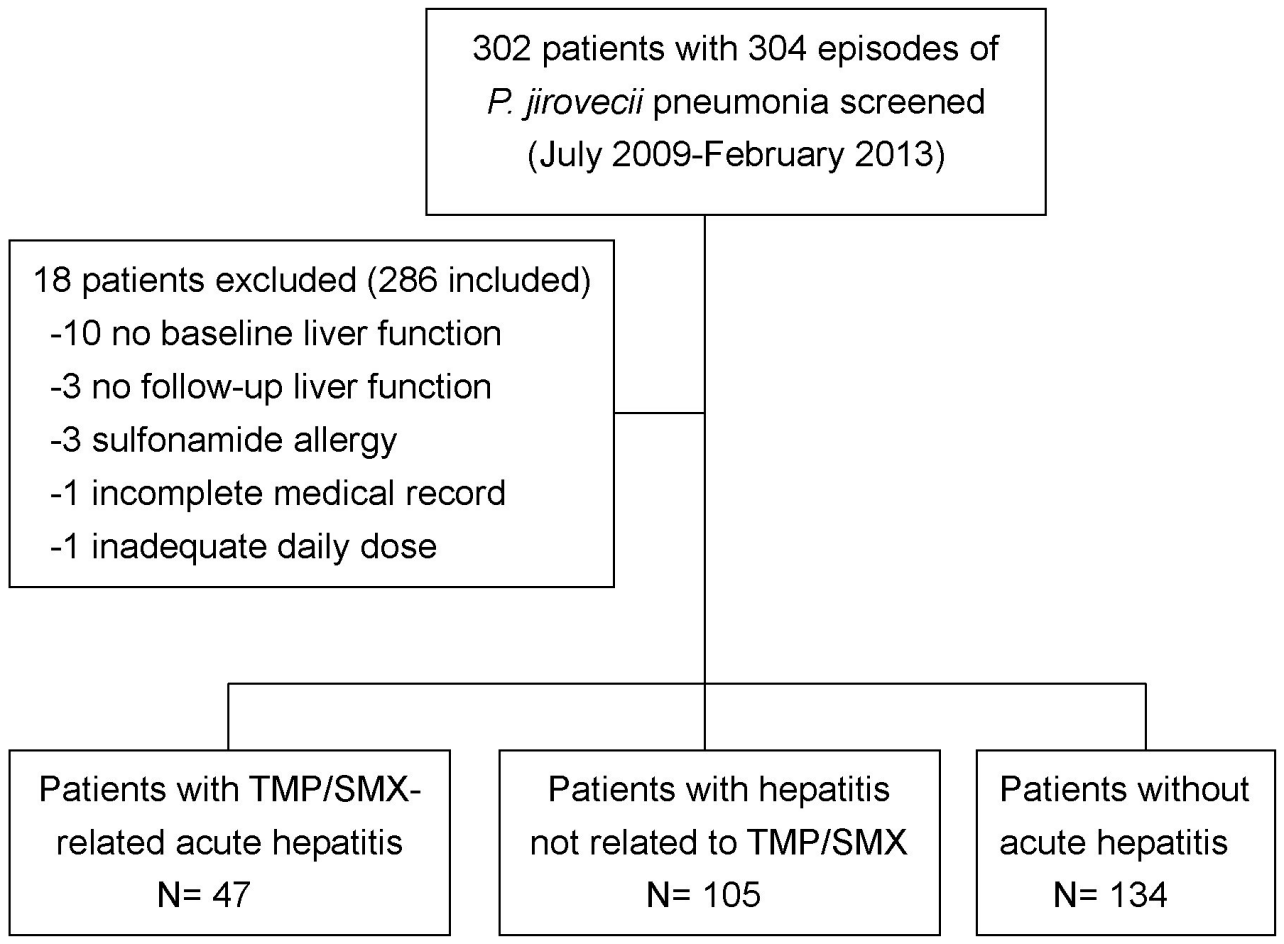
Study flow of HIV-infected patients who developed hepatotoxicity after treatment with trimethoprim/sulfamethoxazole.

During the study period, 302 HIV-infected patients with *P. jirovecii* pneumonia who received TMP/SMX were enrolled (Figure 1). Overall, 18 patients (6.0%) were excluded: 3 with sulfonamide allergy; 10 without liver-function test results at baseline; 3 without follow-up liver-function tests after initiation of TMP/SMX; 1 without a complete medical record; and 1 receiving a suboptimal daily dose of TMP/SMX. Of the 286 treatment courses that administered to 284 patients, 152 cases of hepatotoxicity (53.1%) were detected and 47 (16.4%) were classified as TMP/SMX-related hepatotoxicity by RUCAM scale (Figure 1 and Table S1). Of those 47 cases of TMP/SMX-related hepatotoxicity, 42 (89.4%) were classified as hepatocellular type, 1 (2.1%) cholestatic type, and the other 4 (8.5%) mixed type. Twenty of the cases (42.6%) were of grade 3 to 4 hepatotoxicity, and 3 (6.4%) developed jaundice. The interval between initiation of TMP/SMX and development of hepatotoxicity ranged from 2 to 24 days (median, 11 days). Among the 47 patients, 41 (87.2%) had normalization of liver-function profiles after dose reduction or discontinuation of TMP/SMX. Twenty patients (42.6%) had to discontinue TMP/SMX and change to other regimens (clindamycin plus primaquine or dapsone), and 16 (34.0%) had to reduce the dose of TMP/SMX; the other 11 patients had been treated with TMP/SMX for at least 10 days, and decisions to discontinue TMP/SMX were made by the treating physicians.

There were 15 (31.9%) and 28 patients (20.9%) in the hepatotoxicity group and control group (patients without hepatotoxicity), respectively, that required respiratory support in the intensive care unit (*P* = 0.13); 4 (8.5%) and 12 patients (9.0%) of the respective group died within 30 days of hospitalization (*P* = 0.999). The median length of hospital stay for the hepatotoxicity and control group was 18 days (interquartile range [IQR], 12–27) and 15 days (IQR, 10–27), respectively (*P* = 0.17). None of them developed acute liver failure with coagulopathy, and none of the deaths resulted from hepatotoxicity.

Table 1 shows the clinical characteristics of the patients in the control group (n = 134 episodes) and those (47) with TMP/SMX-related hepatotoxicity. Compared with the patients in the control group, patients with hepatotoxicity had a higher BMI (20.3 vs 19.5, *P* = 0.070), were less likely to have previous exposure to antiretroviral therapy (8.5% vs 28.4%, *P* = 0.006), and were more likely to have diabetes mellitus (10.6% vs 2.2%, *P* = 0.029). However, both groups had similar daily dosage of TMP/SMX (median dose, 14.2 vs 14.4 mg/kg/day, *P* = 0.479).

**Table 1:** Clinical characteristics of HIV-infected patients with and those without hepatotoxicity after receiving trimethoprim/sulfamethoxazole for treatment of *Pneumocystis jirovecii* pneumonia.

Characteristic	Total (n = 181)	Hepatotoxicity group (n = 47)	Control group (n = 134)	*P* value
Age, median (range), years	34.9 (19–81)	34.17 (19–81)	34.88 (19–78)	0.562
Male sex, N (%)	177 (97.8)	47 (100)	130 (97.0)	0.574
Weight, kg	56 (33–93)	58 (42–93)	55.9 (33–93)	0.134
BMI, kg/m^2^	19.9 (13.3–35.9) [N = 178]	20.3 (15.3–35.9) [N = 47]	19.5 (13.0–30.8) [N = 131]	0.070
Risk Factors				
MSM	136 (75.1)	40 (76.6)	100 (74.6)	0.700
Heterosexual	29 (16.0)	5 (10.6)	24 (17.9)	0.242
IDU	5 (2.8)	2(4.3)	3 (2.2)	0.606
Unknown	12 (6.6)	4 (8.5)	8 (6.0)	0.512
Smoking	83 (29.0)	23 (48.9)	60 (44.8)	0.622
Alcohol use	22 (12.6) [N = 174]	5 (10.64)	17 (13.39) [N = 127]	0.628
Prior exposure to antiretrovirals	42 (23.2)	4 (8.5)	38 (28.4)	0.006
Prior exposure to TMP/SMX	17 (9.4)	3 (6.4)	14 (10.5)	0.565
Other medical diseases				
Diabetes mellitus	8 (4.4)	5 (10.6)	3 (2.2)	0.029
Hypertension	2 (1.1)	1 (2.1)	1 (0.75)	0.453
Chronic lung disease	19 (10.5)	5 (10.6)	14 (10.5)	0.999
Chronic kidney disease	17 (9.4)	3 (6.4)	14 (10.5)	0.565
Malignancy	5 (2.8)	0 (0)	5 (3.7)	0.329
Tuberculosis	4 (2.2)	2 (4.3)	2 (1.5)	0.277
HBsAg-positive	36 (20.6) [N = 175]	9 (19.6) [N = 46]	27 (20.9) [N = 129]	0.844
Anti-HCV-positive	7 (4.1) [N = 173]	1 (2.2) [N = 46]	6 (4.7) [N = 127]	0.677
Baseline laboratory data at the start of TMP/SMX median(range)				
Creatinine, mg/dL	0.84 (0.22–2.07)	0.88 (0.54–2.0)	0.84 (0.22–2.07)	0.224
AST (U/L)	43 (11–820) [N = 154]	43.5 (11–277) [N = 42]	43 (12–820) [N = 113]	0.861
ALT (U/L)	24 (5–354) [N = 170]	26.5 (9–116) [N = 44]	23 (5–354) [N = 126]	0.472
Total bilirubin, mg/dL	0.56 (0.14–17.32) [N = 115]	0.6 (0.14–1.28) [N = 35]	0.6 (0.16–17.32) [N = 80]	0.385
ALP (U/L)	116 (21–786) [N = 68]	117 (38–431) [N = 24]	114 (21–786) [N = 44]	0.908
LDH (U/L)	636 (133–3442) [N = 115]	645 (204–2258) [N = 35]	627 (133–3442) [N = 80]	0.512
*NAT1* (slow acetylator)	5 (8.77) [N = 57]	2 (10) [N = 20]	3 (8.11) [N = 37]	0.810
*NAT2* (slow acetylator)	27 (35.5) [N = 76]]	9 (36) [N = 25]	18 (35.3) [N = 51]	0.952

**Note:** Data represent the median value (range) for continuous variables and the number of cases (%) for categorical variables. N indicates the number of patients being tested.

**Abbreviations:** AST, aspartate aminotransferase; ALT, alanine aminotransferase; ALP, alkaline phosphatase; BMI, body-mass index; HBsAg, hepatitis B virus surface antigen; HCV, hepatitis C virus; IDU, injecting drug user; LDH, lactate dehydrogenase; TMP/SMX, trimethoprim/sulfamethoxazole; *NAT1*, *N*-acetyltransferase-1; *NAT2*, *N*-acetyltransferase-2.

In total, 96 and 128 of the 141 patients enrolled at the National Taiwan University Hospital had data on *NAT1* and *NAT2* genes, respectively. The proportion of patients with *NAT1* and *NAT2* genes predictive of slow acetylation phenotype was not statistically significantly different between the patients who developed TMP/SMX-related hepatotoxicity and those who did not (Table 1).

Concomitant medications and treatment responses of *P. jirovecii* pneumonia to TMP/SMX are shown in Table 2. Compared with patients without hepatotoxicity, patients with hepatotoxicity had a higher CD4 count at the start of TMP/SMX (median, 48 vs 29 cells/µL, *P* = 0.083), were less likely to have concomitant use of cART (55.3% vs 73.1%, *P* = 0.031) and fluconazole (51.1% vs 61.9%, *P* = 0.191), and were more likely to develop respiratory failure (29.8% vs 18.7%, *P* = 0.110) in univariate analysis.

**Table 2:** Virologic, immunologic and clinical status of HIV infection and treatment of HIV-infected patients with and those without hepatotoxicity after receiving trimethoprim/sulfamethoxazole.

Characteristic	Total (n = 181)	Hepatotoxicity group (n = 47)	Control group (n = 134)	*P* value
Plasma HIV RNA load at the start of TMP/SMX log_10_ copies/mL	5.24 (2.05–6.94) [N = 173]	5.09 (2.27–6.39) [N = 46]	5.24 (2.05–6.94) [N = 127]	0.873
CD4 count at the start of TMP/SMX, cells/µl	34 (1–440) [N = 180]	48 (3–173)	29 (1–440) [N = 133]	0.083
Plasma CMV viral load at the start of TMP/SMX, log_10_ copies/mL	3.55 (2.07–5.80) [N = 56]	3.15 (2.34–4.25) [N = 20]	3.60 (2.07–5.80) [N = 36]	0.066
TMP/SMX dose, mg/kg/day	14.4 (4.4–22.2)	14.2 (4.4–22.2)	14.4 (5.3–21.3)	0.479
Use of parenteral TMP/SMX	135 (74.6)	98 (73.1)	37 (78.7)	0.449
Concomitant medications				
cART	123 (68.0)	26 (55.3)	98 (73.1)	0.031
NNRTI*	67 (37.0)	12 (25.5)	55 (41.0)	0.058
PI	57 (31.5)	14 (29.8)	43 (32.1)	0.77
Steroids	102 (56.67) [N = 180]	27 (57.45)	75 (56.39) [N = 133]	0.900
Fluconazole	107 (59.1)	24 (51.1)	83 (61.9)	0.192
Treatment outcome				
ICU admission	43 (23.8)	15 (31.9)	28 (20.9)	0.127
Respiratory failure	39 (21.6)	14 (29.8)	25 (18.7)	0.110
Overall mortality	24 (13.3)	8 (17.0)	16 (11.9)	0.377

**Note:** 1. Data represent the median value (range) for continuous variables and the number of cases (%) for categorical variables. N indicates the number of patients being tested.

2. *Two patients (4.26%) in the hepatotoxicity group and 18 patients (13.43%) in the control group were receiving nevirapine.

**Abbreviations:** CMV, cytomegalovirus; cART, combination antiretroviral therapy; ICU, intensive care unit; NNRTI, non-nucleoside reverse transcriptase inhibitor; PI, protease inhibitor.

Other TMP/SMX-related adverse events are shown in Table 3. Compared with patients without hepatotoxicity, those with hepatotoxicity were more likely to experience acute psychosis (19.6% vs 11.1%, *P* = 0.046), hyperkalemia (36.2% vs 15.8%, *P* = 0.003), and hyponatremia (84.1% vs 66.2%, *P* = 0.024) during the treatment with TMP/SMX in the univariate analysis.

**Table 3:** Other TMP/SMX-associated complications of HIV-infected patients with and those without hepatotoxicity after receiving trimethoprim/sulfamethoxazole.

Characteristics	Total (n = 181)	Hepatotoxicity group (n = 47)	Control group (n = 134)	p value
Acute psychosis^a^	24 (13.3)	9 (19.6)	15 (11.1)	0.046
Hyperkalemia^b^	38 (21.1) [N = 180]	17 (36.2)	21 (15.8) [N = 133]	0.003
Acute kidney injury^c^	20 (11.5) [N = 174]	5 (11.4) [N = 44]	15 (11.5) [N = 130]	0.975
Leukopenia^d^	39 (21.8) [N = 179]	10 (21.3)	29 (22.0) [N = 132]	0.921
Nausea/vomiting	45 (24.9)	11 (23.9)	34 (25.6)	0.915
Skin rash	27 (15.0) [N = 180]	10 (21.3)	17 (12.8) [N = 133]	0.161
Hyponatremia^e^	123 (70.7) [N = 174]	37 (84.1) [N = 44]	86 (66.2) [N = 130]	0.024

**Note:** Data represent the number of cases (%) for categorical variables. N indicates the number of patients being tested. This table includes adverse effects of any grade.

**Definitions:** Acute psychosis was defined as prominent hallucinations or delusions that are judged to be due to the direct physiological effects of a substance. The disturbance must not be better accounted for by a psychotic disorder that is not substance induced, and the diagnosis is not made if the psychotic symptoms occur only during the course of a delirium [7]; hyperkalemia, serum potassium >5.3 mmol/L; acute kidney injury, serum creatinine elevated to more than 0.5 mg/dL compared to the baseline creatinine value; leukopenia, white-blood cell count <4000 cells/µl; and hyponatremia, serum sodium <135 mmol/L.

There were no statistically significant differences between the two groups of patients in terms of age, daily dose of TMP/SMX, serostatus of hepatitis B or C virus, plasma HIV RNA load, alcohol ingestion, smoking, use of steroids, baseline total bilirubin, ALT, AST, and LDH level, mortality, development of skin rash, nausea or vomiting, and leukopenia.

In logistic regression analysis (Table 4), concomitant use of fluconazole for oro-esophageal candidiasis was significantly associated with a lower risk of hepatotoxicity (adjust odds ratio, 0.372; 95% confidence interval, 0.145 to 0.957, *P* = 0.040). If we included only those 20 cases of patients with hepatotoxicity of grades 3 and 4, the usage of fluconazole remained a significant protective factor in multivariate logistic regression compared with the control group, with an adjusted odds ratio of 0.262 (95% confidence interval, 0.072–0.951) (Table S2).

**Table 4:** Multivariate logistic regression for the factors associated with trimethoprim/sulfamethoxazole-related hepatotoxicity.

Factors	Adjusted odds ratio	95% Confidence interval	*P* value
BMI	1.068	0.951–1.20	0.271
Diabetes mellitus	1.001	0.994–1.008	0.768
CD4 count at the start of TMP/SMX, per 1 cell/µL increase	1.002	0.994–1.009	0.676
Baseline total bilirubin levels, per 1 mg/dL increase	0.482	0.162–1.439	0.191
Concomitant use of fluconazole	0.372	0.145–0.957	0.040
Previous exposure to antiretroviral therapy	0.4	0.109–1.464	0.166
Hyperkalemia	2.608	0.938–7.254	0.066
Psychosis	1.468	0.513–4.20	0.474

**Note:** Data represents the point estimate of the odds ratio for developing hepatotoxicity with 95% confidence interval.

**Abbreviations:** BMI, body-mass index (kg/m^2^); TMP/SMX, trimethoprim/sulfamethoxazole.

The results of comparisons between the patients with all-cause hepatotoxicity and those without hepatotoxicity in multivariate logistic regression analysis are shown in supplementary table 3, in which a heavier weight, being antiretroviral-naive, no concomitant use of ritonavir, and respiratory failure during the treatment course were associated with a higher risk of hepatotoxicity.

**Figure 2:**
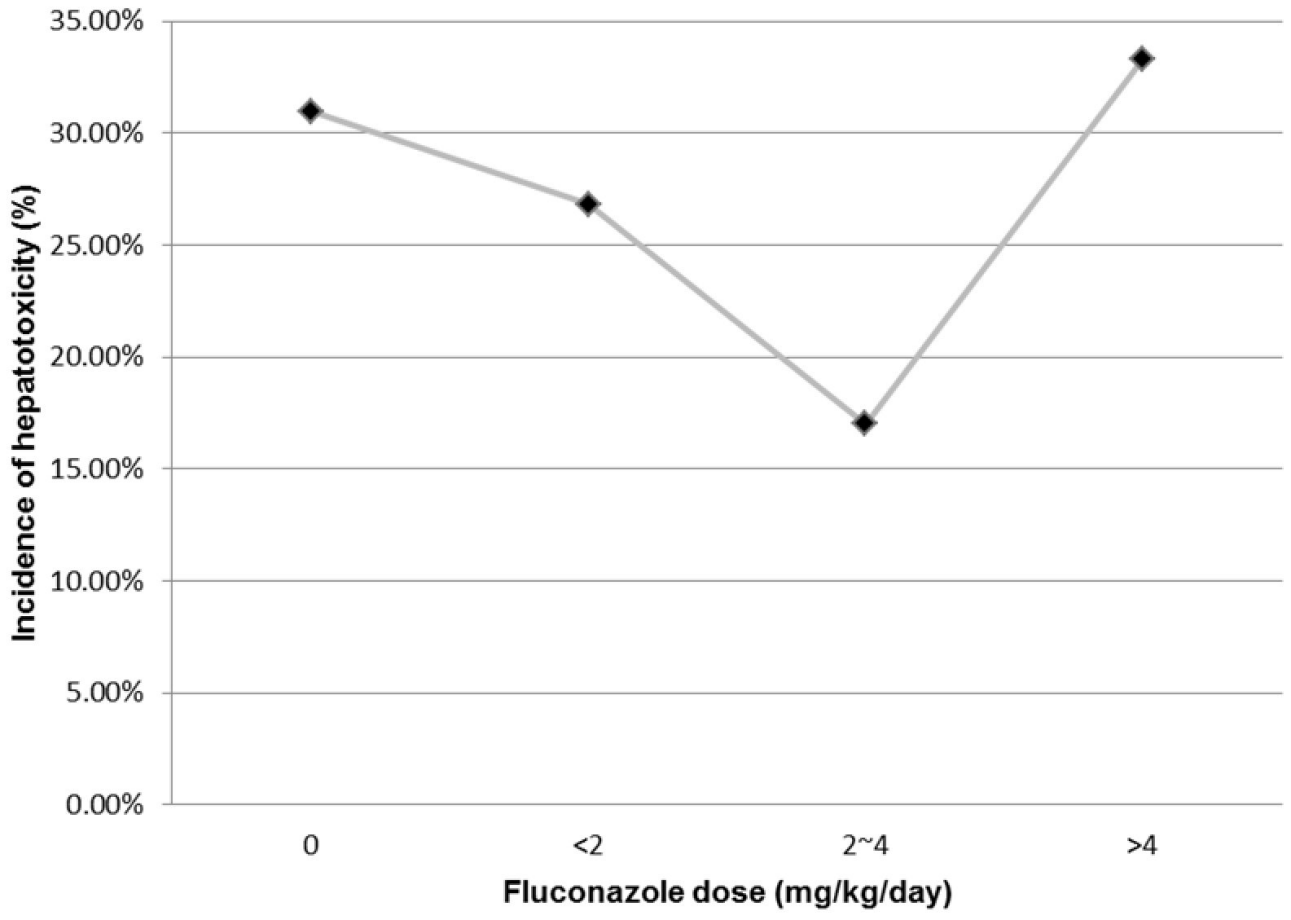
Trends of incidence of trimethoprim/sulfamethoxazole-related hepatotoxicity (Y-axis) and daily dose of fluconazole in mg/kg (X-axis) (*P* for trends, 0.343).

The incidence of TMP/SMX-related hepatotoxicity decreased with an increasing daily dose of fluconazole until the daily dose reached 4 mg/kg (Figure 2). After excluding those patients who received fluconazole at a daily dose of greater than 4 mg/kg, the protective role of fluconazole in TMP/SMX-related hepatotoxicity was accentuated(adjusted odds ratio, 0.263; 95% confidence interval, 0.096 to 0.718; *P* = 0.009) (data not shown). Similar trends were also observed when all of the 152 cases of hepatotoxicity were included (Figure S1).

## Discussion

In this multicenter, retrospective study, we found that the estimated incidence of TMP/SMX-related hepatotoxicity in HIV-infected Taiwanese patients who received TMP/SMX for *P. jirovecii* pneumonia was 16.4%. Hepatotoxicity was mainly of the hepatocellular type and concomitant use of fluconazole for candidiasis attenuated the risk of hepatotoxicity.

The incidence of TMP/SMX-related hepatotoxicity observed in this study in the HIV-infected patients with *P. jirovecii* pneumonia is higher than that observed in several randomized clinical trials that compared the efficacy between TMP/SMX and other regimens in western countries [22]–[25]. In those studies, the incidence of TMP/SMX-related hepatotoxicity was consistently lower than 10%. The discrepancy in the incidence of hepatotoxicity may be explained by the differences in populations studied, concurrent medications, and criteria used in defining hepatotoxicity. The higher rate of hepatotoxicity in the current study might be because we used 2-fold or greater increase of aminotransferase or total bilirubin level from baselines to define hepatotoxicity and inclusion of some patients who continued TMP/SMX at a lower dose. If we use 5-fold or greater increase of aminotransferase levels (grade 3 or higher) to define hepatotoxicity, the incidence would be 7.0% in our study.

**Figure 3:**
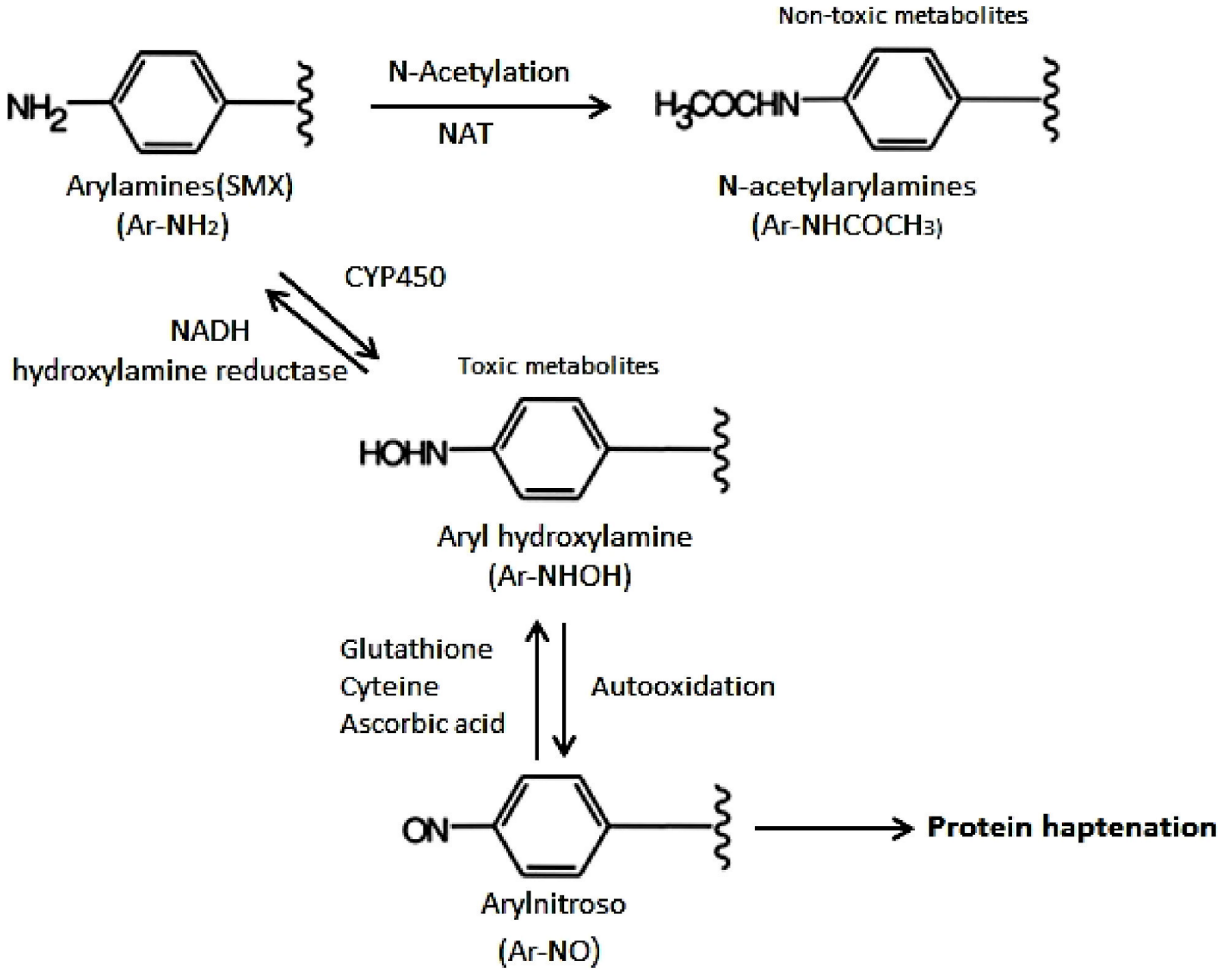
Metabolic pathways of sulfamethoxazole.

TMP/SMX-induced hepatotoxicity may occur as a result of two postulated mechanisms (Figure 3) [26]. Hypersensitivity to TMP/SMX possibly mediated by glutathione metabolism may cause hepatotoxicity [10], [27], [28]. Wang et al found SNP rs761142 in the glutamate cysteine ligase catalytic (GCLC) subunit to be associated with reduced GCLC mRNA expression. By catalyzing a critical step in glutathione biosynthesis, it was associated with SMX-induced hypersensitivity in HIV-infected patients [29].

The metabolites of TMP/SMX may also cause hepatocellular damage or cholestasis. Hepatic *N*-acetyltransferase plays a role in transforming TMP/SMX to non-toxic metabolites, and, therefore, the rate of acetylation may contribute to the risks of TMP/SMX-related hepatotoxicity. A previous study by Smith et al has shown that HIV-infected patients with slow acetylation phenotypes had a higher incidence of adverse reactions to TMP/SMX [30]. In an ethnic Chinese population, the frequency of the slow acetylation genotypes is about 25% [31], whereas it is above 50% in Caucasians. Hence the acetylation phenotype cannot explain the higher rates of hepatotoxicity observed among HIV-infected ethnic Chinese in our study compared to that previously reported by studies conducted in western countries.

The alternative pathway of TMP/SMX metabolism is via the cytochrome protein 450 (CYP) 2C9 pathway, wherein sulfamethoxazole is metabolized to hydroxylamine by the CYP 2C9 pathway. Hydroxylamine plays an important role in hepatotoxicity [32]–[34]. The frequency of poor metabolizers for CYP 2C9 in the Taiwanese population is about 8.2%, which is significantly lower than that for Caucasians (about 20%) [35]. Therefore, the likelihood of TMP/SMX being metabolized rapidly to hydroxylamine will be higher among Taiwanese, which may contribute to the higher incidence of hepatotoxicity observed in our study.

Fluconazole is a potential inhibitor of CYP2C9 activity [36], [37]. In a previous pharmacokinetic study in healthy volunteers who concurrently received fluconazole and TMP/SMX, Mirta et al demonstrated that use of fluconazole decreased the area-under-the-curve (AUC) of hydroxylamine by 37% [38]. Therefore, we postulate that our findings of reduced risk of TMP/SMX-related hepatotoxicity in association with concomitant use of fluconazole may be explained by reduced formation of hydroxylamine by fluconazole, though more pharmacologic studies are warranted to confirm our hypothesis.

Several previous reports have shown that fluconazole is in itself hepatotoxic in a dose-dependent manner [39]–[41]. The incidence of hepatotoxicity during the treatment course of fluconazole was approximately 10%, and only 0.7% of the patients needed discontinuation of therapy due to severe hepatotoxicity. The liver function profiles generally recovered after discontinuing or tapering the dose of fluconazole [42], [43]. Given the intrinsic hepatotoxicity potential of fluconazole and inhibition of CYP2C9 by fluconazole that may provide protective effects in patients receiving TMP/SMX for *P. jirovecii* pneumonia, it is not clear with respect to the appropriate dose of fluconazole that may balance the risk of potentiation of hepatotoxicity with protective effects. As shown in Figure 2 and Figure S1, the incidence of TMP/SMX-related hepatotoxicity decreased from 31% in patients not receiving concomitant fluconazole to 17% in those receiving fluconazole at a daily dose of 2–4 mg/kg, but the trends reversed when the daily fluconazole dose increased to greater than 4 mg/kg in the patients we studied. While we do not have good explanation why the trends reversed with higher doses of fluconazole than 4 mg/kg, the potential risk of hepatotoxicity with further increase of fluconazole doses may exceed the potential benefit conferred by inhibition of formation of hepatotoxic hydroxylamine by CYP2C9.

Antiretroviral therapy, especially nevirapine, could cause hepatotoxicity [44]. However, fewer patients in the hepatotoxicity group had a history of previous antiretroviral exposure or were concurrently taking antiretroviral therapy when TMP/SMX was continued, which suggests that antiretroviral therapy might play a minor role, if any, in hepatotoxicity detected in those patients with TMP/SMX-related hepatotoxicity.

There are several limitations of our study and interpretation of our results should be cautious. First, this is a retrospective study and the timing and frequency of follow-up of liver-function profiles were not standardized, which may preclude us from knowing exactly when TMP/SMX-related hepatotoxicity developed. Second, while we recorded alcohol ingestion, the amount of alcohol consumed on a daily basis and the use of concurrent medications that may cause hepatotoxicity such as statins was not recorded. In our study population, the mean age was 34 years, and, only 8.5% in the hepatotoxicity group had previous exposure to antiretroviral therapy (Table 1), rendering the use of lipid-modifying agents for cART-related dyslipidemia uncommon. Third, 105 patients with hepatotoxicity were excluded from analysis and all the patients were HIV-infected ethnic Chinese, hence the findings may not be generalizable to patients of other ethnicities or non-HIV-infected patients who may have different pharmacokinetic and pharmacogenetic profiles. Fourth, the tests for acetylator types were only performed in a subgroup of patients. Therefore, the exact role of *NAT* genes could not be appropriately examined in our study. Fifth, while we used the RUCAM scale to increase the reproducibility of our findings, misclassifications may still occur because the cause of elevated aminotransferase levels in the HIV-infected patients are often multifactorial. However, a recent study has shown that RUCAM scale had similar accuracy, but better reproducibility (agreement between the observers) than the NARANJO scale [45], though the bias of RUCAM scale utilization should still be considered. Sixth, none of our patients underwent liver biopsy. Non-alcoholic steatohepatitis (NASH) is commonly associated with abdominal obesity and metabolic disorders for HIV-infected patients, and it could evolve to severe liver injuries including nonalcoholic cirrhosis and hepatocellular carcinoma [46]. Last, the serum concentrations of TMP/SMX and its metabolites such as hydroxylamine were not measured and the potential drug-drug interactions between TMP/SMX and antiretrovirals were not examined in our study population. However, of all antiretrovirals, only efavirenz may inhibit CYP 2C9, which may decrease the concentration of hydroxylamine [47]. In both groups, the proportions of patients who subsequently received efavirenz while receiving TMP/SMX were similar (21.3% vs 26.9%, *P* = 0.449) (data not shown).

In conclusion, we found that the incidence of TMP/SMX-related hepatotoxicity was estimated 16.4% among HIV-infected patients who are ethnic Chinese and received TMP/SMX for treatment of *P. jirovecii* pneumonia. Concomitant use of fluconazole at a dose not exceeding 4 mg/kg was associated with reduced risk for TMP/SMX-related hepatotoxicity.

## Supporting Information

Figure S1 **Trends of the incidence of trimethoprim/sulfamethoxazole-related hepatotoxicity (Y-axis) and daily dose of fluconazole in mg/kg (X-axis) for all 286 cases.**
(DOC)

Table S1 Clinical characteristics of the 286 HIV-infected patients who received trimethoprim/sulfamethoxazole for treatment of *Pneumocystis jirovecii* pneumonia.
(DOC)

Table S2 Multivariate logistic regression for the possible factors associated with all-cause hepatotoxicity in HIV-infected patients receiving trimethoprim/sulfamethoxazole for treatment of *Pneumocystis jirovecii* pneumonia.
(DOC)
