# Supplementary material for: Multicenter Study of Trimethoprim/Sulfamethoxazole-Related Hepatotoxicity: Incidence and Associated Factors among HIV-Infected Patients Treated for Pneumocystis jirovecii Pneumonia
Source: PLoS One. 2014 Sep 3;9(9):e106141. doi: 10.1371/journal.pone.0106141 (PMC4153565; doi:10.1371/journal.pone.0106141)

**Supporting Information**

**Figure S1.** Trends of incidence of TMP/SMX-related hepatotoxicity (Y-axis) and daily dose of fluconazole in mg/kg (X-axis), with all 286 cases included


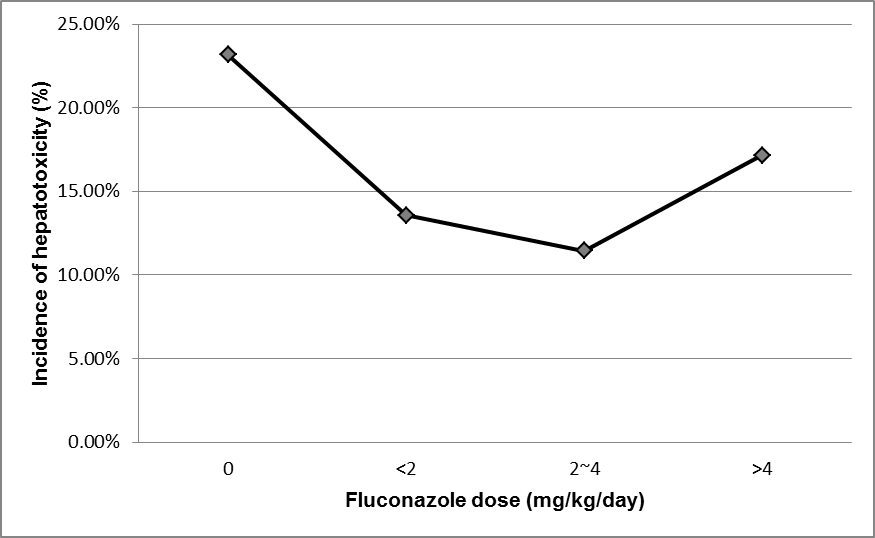

Supplement: Figure S1 — Trends of the incidence of trimethoprim/sulfamethoxazole-related hepatotoxicity (Y-axis) and daily dose of fluconazole in mg/kg (X-axis) for all 286 cases. (DOC) [file pone.0106141.s001.doc]
