# Supplementary material for: Multicenter Study of Trimethoprim/Sulfamethoxazole-Related Hepatotoxicity: Incidence and Associated Factors among HIV-Infected Patients Treated for Pneumocystis jirovecii Pneumonia
Source: PLoS One. 2014 Sep 3;9(9):e106141. doi: 10.1371/journal.pone.0106141 (PMC4153565; doi:10.1371/journal.pone.0106141)
Supplement: Table S1 — Clinical characteristics of the 286 HIV-infected patients who received trimethoprim/sulfamethoxazole for treatment of Pneumocystis jirovecii pneumonia. (DOC) [file pone.0106141.s002.doc]

**Supporting Information**

| **Table S1.** Clinical characteristics of the 286 HIV-infected patients who received trimethoprim/sulfamethoxazole for treatment of *Pneumocystis jirovecii* pneumonia | | | | | | | | | |
| --- | --- | --- | --- | --- | --- | --- | --- | --- | --- |
| Characteristic | Total (n=286) | | | All-cause hepatotoxicity group (n=152) | | | | Control group (n=134) | *P* value |
| Age, years | 35.1 (18-81) | | | 35.8 (18-81) | | | | 34.88 (19-78) | 0.730 |
| Male sex | 281 (98.25) | | | 151 (99.34) | | | | 130 (97.01) | 0.574 |
| Weight, kg | 57 (33-109) [N=285] | | | 58 (37-109) | | | | 55.9 (33-93) | 0.042 |
| BMI | 19.8 (13.3-35.9) [N=279] | | | 20.1 (13.3-35.9) [N=148] | | | | 19.5 (13.2-30.8) [N=131] | 0.141 |
| HIV Risk Factors |  | | |  | | | |  |  |
| homosexual | 199 (69.58) | | | 108 (71.05) | | | | 91 (67.91) | 0.564 |
| heterosexual | 40 (13.99) | | | 16 (10.53) | | | | 24 (17.91) | 0.072 |
| bisexual | 19 (6.64) | | | 10 (6.58) | | | | 9 (6.72) | 0.963 |
| IDU | 12 (4.20) | | | 9 (5.92) | | | | 3 (2.24) | 0.121 |
| Unknown | 18 (6.29) | | | 10 (6.58) | | | | 8 (5.97) | 0.833 |
| Smoking | 118 (41.26) | | | 63 (41.45) | | | | 60 (44.78) | 0.398 |
| Alcohol Use | 32 (11.55) [N=277] | | | 15 (10) [N=150] | | | | 17 (13.39) [N=127] | 0.380 |
| Prior exposure to antiretrovirals | 65 (22.73) | | | 27 (17.76) | | | | 38 (28.36) | 0.064 |
| Prior exposure to TMP-SMX | 29 (10.14) | | | 15 (9.87) | | | | 14 (10.53) | 0.979 |
| Other medical diseases | | | | | | | |  |  |
| Diabetes mellitus | | | 8 (2.80) | | 5 (3.29) | | | 3 (2.24) | 0.727 |
| Hypertension | | | 4 (1.40) | | 3 (1.97) | | | 1 (0.75) | 0.625 |
| Chronic lung disease | | | 29 (10.14) | | 15 (9.87) | | | 14 (10.45) | 0.871 |
| Chronic kidney disease | | | 20 (6.99) | | 6 (3.95) | | | 14 (10.45) | 0.032 |
| Malignancy | | | 6 (2.10) | | 1 (0.66) | | | 5 (3.73) | 0.102 |
| Tuberculosis | | | 8 (2.80) | | 6 (3.95) | | | 2 (1.49) | 0.290 |
| HBsAg-positive | | | 66 (23.91) [N=276] | | 39 (25.85) [N=147] | | | 27 (20.93) [N=129] | 0.421 |
| Anti-HCV-positive | | | 17 (6.14) [N=277] | | 11 (7.38) [N=150] | | | 6 (4.72) [N=127] | 0.352 |
| Baseline blood laboratory data at the start of TMP-SMX | | | | | | | | |  |
| Creatinine (mg/dL) | | 0.82 (0.22-5.68) | | | | 0.81 (0.44-5.68) | 0.84 (0.22-2.07) | | 0.969 |
| AST (U/L) | | 45 (11-2080) [N=244] | | | | 45 (11-2080) [N=131] | 43 (12-820) [N=113] | | 0.540 |
| ALT (U/L) | | 27 (5-2370) [N=271] | | | | 27 (6-2370) [N=145] | 23 (5-354) [N=126] | | 0.057 |
| Total bilirubin (mg/dL) | | 0.58 (0.14-17.32) [N=182] | | | | 0.55 (0.14-4.9) [N=102] | 0.6 (0.16-17.32) [N=80] | | 0.344 |
| ALP (U/L) | | 108.5 (21-786) [N=102] | | | | 98.5 (32-639) [N=58] | 114 (21-786) [N=44] | | 0.570 |
| LDH (U/L) | | 620 (133-3442) [N=178] | | | | 618 (145-2509) [N=97] | 627 (133-3442) [N=80] | | 0.774 |
| NAT1 (slow acetylator) | | 6 (6.52) [N=92] | | | | 3 (5.45) [N=55] | 3 (8.11) [N=37] | | 0.613 |
| NAT2 (slow acetylator) | | 38 (29.7) [N=128] | | | | 20 (26.0) [N=77] | 18 (35.3) [N=51] | | 0.952 |
| **Note:** Data represent the median value (range) for continuous variables and the number of cases (%) for categorical variables. N indicates the number of patients being tested.  **Abbreviations:** AST, aspartate aminotransferase; ALT: alanine aminotransferase; ALP, alkaline phosphatase; BMI, body-mass index; HBsAg, hepatitis B virus surface antigen; HCV, hepatitis C virus; IDU, injecting drug user; LDH, lactate dehydrogenase; TMP/SMX, trimethoprim/sulfamethoxazole; *NAT1*, *N*-acetyltransferase-1; *NAT2*, *N*-acetyltransferase-2 | | | | | | | | | |
