# Supplementary material for: Multicenter Study of Trimethoprim/Sulfamethoxazole-Related Hepatotoxicity: Incidence and Associated Factors among HIV-Infected Patients Treated for Pneumocystis jirovecii Pneumonia
Source: PLoS One. 2014 Sep 3;9(9):e106141. doi: 10.1371/journal.pone.0106141 (PMC4153565; doi:10.1371/journal.pone.0106141)
Supplement: Table S2 — Multivariate logistic regression for the possible factors associated with all-cause hepatotoxicity in HIV-infected patients receiving trimethoprim/sulfamethoxazole for treatment of Pneumocystis jirovecii pneumonia. (DOC) [file pone.0106141.s003.doc]

**Supporting Information**

| **Table S2.** Multivariate logistic regression for the possible factors associated with all-cause hepatotoxicity in HIV-infected patients receiving trimethoprim/sulfamethoxazole for treatment of *Pneumocystis jirovecii* pneumonia | | | |
| --- | --- | --- | --- |
| Factors | Odds ratio | 95% Confidence interval | P value |
| Body weight | 1.024 | 1.001-1.048 | 0.041 |
| Concomitant use of fluconazole | 1.226 | 0.714-2.106 | 0.461 |
| Baseline ALT value at the start of TMP/SMX | 1.001 | 0.999-1.003 | 0.235 |
| CD4 lymphocyte count at the start of TMP/SMX | 0.999 | 0.995-1.003 | 0.577 |
| Previous exposure to antiretrovirals | 0.505 | 0.273-0.933 | 0.029 |
| Acute psychosis | 0.703 | 0.375-1.316 | 0.271 |
| Concomitant use of ritonavir | 0.375 | 0.191-0.737 | 0.005 |
| Respiratory failure | 2.175 | 1.148-4.119 | 0.017 |

**Note:** Data represents the point estimate of the odds ratio for developing hepatotoxicity with 95% confidence interval.

**Abbreviations:** ALT, alanine aminotransferase; BMI, body-mass index (kg/m^2^); TMP/SMX, trimethoprim/sulfamethoxazole
